# Pulse Oximetry Screening Adapted to a System with Home Births: The Dutch Experience

**DOI:** 10.3390/ijns4020011

**Published:** 2018-03-30

**Authors:** Ilona C. Narayen, Nico A. Blom, Arjan B. te Pas

**Affiliations:** 1 Department of Paediatrics, Division of Neonatology, Leiden University Medical Center, P.O. Box 9200, 2300 RC Leiden, The Netherlands; 2 Division of Paediatric Cardiology, Leiden University Medical Center, 2333 ZA Leiden, The Netherlands

**Keywords:** neonates, screening, congenital heart defects, home births

## Abstract

Neonatal screening for critical congenital heart defects is proven to be safe, accurate, and cost-effective. The screening has been implemented in many countries across all continents in the world. However, screening for critical congenital heart defects after home births had not been studied widely yet. The Netherlands is known for its unique perinatal care system with a high rate of home births (18%) and early discharge after an uncomplicated delivery in hospital. We report a feasibility, accuracy, and acceptability study performed in the Dutch perinatal care system. Screening newborns for critical congenital heart defects using pulse oximetry is feasible after home births and early discharge, and acceptable to mothers. The accuracy of the test is comparable to other early-screening settings, with a moderate sensitivity and high specificity.

## 1. Background

To increase the number of timely diagnoses, several studies on screening newborns for critical congenital heart defects (CCHD) using pulse oximetry (PO) have been performed since 2000 and led to an increasing implementation of PO screening across all continents [1,2]. This non-invasive screening method was proven to be reliable, easy to perform, and easy to implement in hospitals. Although studies only investigated the costs, without the long-term benefits, the screening is likely to be cost-effective and studies using questionnaires have shown that the screening was acceptable for parents and caregivers [2,3,4,5]. 

However, all large studies performed so far were in hospital settings and with a postnatal stay of more than five hours. In contrast, The Netherlands has a different perinatal care setting with the highest rate of home births (18%), which are supervised by community midwives [6]. The midwives stay for approximately three hours after birth and come back for their first follow-up visit at day two or three after birth (day of birth is day one). Also, in The Netherlands, mother and newborn are discharged early (within five hours) after uncomplicated vaginal delivery in hospital. For these reasons, the published protocols used in other countries do not match with the Dutch perinatal logistics and it is not possible to extrapolate the results of other PO screening studies to the Dutch perinatal care setting. We therefore performed studies with an adapted PO screening protocol to fit home births and early discharge in the Dutch unique perinatal care setting. 

## 2. Discussion in The Netherlands

After publication of the meta-analysis on PO screening in the Lancet in 2012 it was stated that in The Netherlands it would be difficult to train all 1850 community midwives in performing PO measurements and to provide them all with PO devices [7]. Although the Dutch Association of Pediatricians (NVK) recommends the use of PO in case of resuscitation of a newborn, PO has not been implemented as standard practice in community midwifery [8,9]. The Netherlands has a history of having a high rate of ‘natural’ deliveries at home, without medical intervention [10]. Community midwives in The Netherlands are traditionally trained in clinical assessment and intervention with little use of technical devices [9]. However, in the Leiden region there is a well-organized clinical and research collaboration between hospitals and community midwives. The midwives participated in a study with recording PO measurements at birth at home. The midwives were trained in one afternoon session and experienced no problems with the use of PO during the study. The study showed that using the PO at home birth was feasible and almost all midwives were enthusiastic about having a PO available, especially in situations with a suboptimal condition of the newborn [9]. We considered the Leiden region the optimal region to pilot PO screening in the Dutch perinatal care setting. 

The screening protocol used in the United States and Scandinavia needed to be adapted and made to fit with the visiting scheme of community midwives in The Netherlands [10,11]. Instead of performing one pre- and post-ductal SpO_2_ reading 24–48 h after birth, we decided to perform these measurements at two separate time points: the first measurement at least one hour after birth, and the second measurement on day two or three of the newborn’s life (day of birth is day one). The first measurement should be performed in the first hours after birth, since community midwives stay for approximately three hours after a delivery and because of discharge within five hours after in-hospital delivery. We were aware that performing screening early (before 24 h) is accompanied with a higher false positive rate due to transitional circulation [2]. However, studies also demonstrated that when the screening was performed after 24 h of life, some CCHD already presented with severe symptoms before the screening was performed [5,12]. The intention of screening is to detect pathology before symptoms occur, making early screening pivotal. Early screening also enables timely detection of other significant pathology, such as infections and respiratory morbidity. We added the second measurement on day two or three of life using the same protocol, at the first follow-up visit of the community midwife, because it is possible that a widely patent ductus arteriosus can cause normal SpO_2_ values in newborns with CCHD in the first hours of life. Midwives and nurses were trained in performing and interpreting the PO measurements in a one-day education session. A web-based entry form with an automatic algorithm interpretation was used for quality assurance, and the research team could be reached 24/7 for questions regarding the screening. Handheld pulse oximeters were used for both studies, supplied by Medtronic (Dublin, Ireland). Reusable sensors were used in order to reduce the costs.

## 3. Pilot Study

We first piloted the adapted protocol in a feasibility study in the Leiden region, in which one academic hospital, two regional hospitals, and 14 midwifery practices are situated [13]. In this study, the Pulse Oximetry Leiden Screening (POLS) study, screening could only be performed after parental consent. Almost all parents who were approached consented and 99% (3059/3090) of the newborns with parental consent were screened. It was reassuring to observe that during the first screening episode in most of the healthy term newborns the pre- and post-ductal SpO_2_ was already above 95% in the first hours after birth (10th percentile was 97% pre-ductally and 96% post-ductally within the first hour after birth, *n* = 394). This implies that newborns with SpO_2_ values below 95% should be evaluated when they are measured at least one hour after birth. Indeed, in 50% of the newborns with a false positive screening result other morbidities than CCHD were diagnosed, including infections, wet lungs, PPHN, or non-critical congenital heart defects. 

## 4. Acceptability to Mothers

We then assessed the acceptability of performing PO screening at home amongst 1172 mothers participating in the feasibility study by using questionnaire [14]. In this group, screening measurements were performed at least once at home by their community midwife. The response rate was acceptable (77%) and the vast majority (93%) of mothers considered the screening test important for all babies and would recommend the test to someone else. 

We concluded that PO screening for CCHD, using the adapted protocol, was feasible in the Dutch perinatal care setting and that screening at home is acceptable to mothers [13,14]. 

## 5. Accuracy Study

In order to assess the accuracy of the adapted PO screening, we performed an implementation study in a larger cohort in a much larger region (Leiden–Amsterdam Region (POLAR) study) [15]. This study was carried out in three academic hospitals, 11 regional hospitals and 75 midwifery practices and included 23,959 newborns. The prenatal detection rate was 73% and the sensitivity was 50%, with a specificity of 99%. Four out of five detected CCHD were identified in the first screening test, in the first hours after birth. The fifth CCHD was diagnosed at 12 h after birth, on the day after the date of birth (day 2 of life). Serious illnesses such as infections and respiratory pathology were detected in 61% of all newborns with false positive screening results (Table 1). This study demonstrated that PO screening adapted to home births and early post-delivery hospital discharge contributes to the detection of CCHD in an early, asymptomatic stage. The early detection of CCHD, but also other significant pathologies, such as infections and respiratory morbidity, could be considered as a safety net when newborns are born at home or early discharged after delivery in hospital. In that view, the PO screening has the potential to decrease morbidity and mortality of newborns in The Netherlands.

## 6. Costs

Before screening programs can be recommended for universal implementation, cost effectiveness should be considered. Cost analyses have shown that the PO screening is likely to be cost effective, but only screening in hospitals was taken into account [3,4,16]. The outcome of children after pediatric cardiac surgery has considerably improved in the last decades, but recent data on gained quality-adjusted life years (QALYs) are lacking. However, it is known that a timely diagnosis of CCHD decreases the risk of mortality and morbidity, and also the length of hospital stay [17,18]. 

In the way our screening was set up, all community midwives would require a pulse oximeter, and positive screenings at home should be transported and referred to hospital. This is likely to increase the costs when performing the screening in the Dutch perinatal care system as compared to settings with deliveries and screening in hospital. Therefore, we also performed a cost-effectiveness analysis with the results of the implementation study. These results will also be published in another article. 

## 7. Prenatal Detection and Sensitivity of PO Screening

PO screening is not a replacement for other screening methods for CCHD, but should be considered as an addition to prenatal screening and physical examination. An early prenatal diagnosis of CCHD allows the parents to be mentally prepared, and gives them the opportunity to terminate the pregnancy. Furthermore, it allows the medical team to prepare a treatment strategy and the delivery can be planned in a congenital heart disease center with a level 3 NICU facility to enable acute surgical or catheter interventions. Prenatal detection varies between countries, and regions within countries, and can be improved with training and logistic interventions [19]. The sensitivity of PO screening is correlated with the prenatal detection rate of CCHD, which ranged from 0 to 82% within performed accuracy studies [20]. Fetal screening, which includes structural anomaly scans, is well organized and highly accessible in The Netherlands; there are strict nationwide requirements regarding the performance of the fetal ultrasounds. Intensive training and audit programs are regionally organized. The prenatal detection rate of CCHD was high in the region where the implementation study was performed [15], but the prenatal detection rate in the other regions of The Netherlands is currently unknown.

Although the overall prenatal detection of CCHD is high, specific defects remain difficult to detect prenatally, such as transposition of the great arteries (TGA), total anomalous pulmonary venous return (TAPVR), pulmonary valve stenosis, aortic valve stenosis, and coarctation of the aorta (CoA) [19,21]. PO screening is efficient at detecting low SpO_2_ caused by TGA, TAPVR, and pulmonary valve stenosis, but left-sided obstructive lesions—such as CoA—are frequently missed with PO screening [2,22,23]. It remains challenging to detect CoA in an early stage even in combination with antenatal screening, PO screening and neonatal physical examination. In conclusion, PO is an effective screening method for identifying CCHD, but results of PO screening are correlated with the prenatal detection rate of CCHD. When considering the implementation of PO screening in The Netherlands and anticipating a variable prenatal detection rate in the Dutch regions, the sensitivity is likely to be somewhere between 50% and 70% [15].

## 8. Comparison with Other Studies

Several studies on PO screening in hospitals were performed which led to implementation in many countries. We performed the first studies, including a feasibility study and a large implementation study, with an adapted protocol for PO screening in a perinatal care system with home births and early postnatal discharge from hospital. Smaller pilot studies on PO screening out-of-hospital settings were performed in the United Kingdom (*n* = 90) and in the plain community in Wisconsin (*n* = 440) [24,25]. In The Netherlands, only women with low-risk pregnancies can choose to have a home birth, while in the Plain community in Wisconsin place of birth is not selected based on a risk profile. Instead it is culturally, religiously or financially based and many pregnant women in the Plain community do not receive prenatal screening. The detection of CCHD in this group will probably be higher when compared to our population of home birth deliveries.

This was the first screening set up where two separate screening episodes were used. Also, the first screening moment was earlier when compared to other early screening studies (8, 30). In general, it is not recommended to perform PO screening in the first hours after birth, because of the probability of having a higher false positive rate due to transitional circulation. In our Leiden pilot study, however, we demonstrated that SpO_2_ values in healthy newborns were above 95% within the first hour of life [13].

## 9. Current Status

After finalizing the studies, a large number of caregivers did not want to await a governmental decision regarding top-down universal implementation, which can take several years. Bottom-up implementation has already begun in the studied region using the logistics that was set up for the study; the screening is continued in all participating hospitals in the POLAR study, as well as by 36% of all participating community midwifery practices, and this rate is still increasing. The perinatal caregivers in these hospitals and practices were convinced of the usefulness of PO screening.

## 10. Conclusions

PO screening for CCHD is feasible to perform and acceptable to mothers in the Dutch perinatal care setting with an adapted protocol for home births and early postnatal discharge from hospital. The screening detects CCHD at an early symptomatic stage with the extra benefit of detecting other significant and potentially life-threatening morbidities, such as infections and respiratory pathology. Implementation of PO screening for CCHD and other morbidities has the potential to decrease infant morbidity and mortality and increase the safety of newborns born at home or discharged from hospital in the first hours of life.

## Figures and Tables

**Table 1:** Overview of screening parameters for pilot and accuracy study

	Pilot Study (*n* = 3059)	Accuracy Study (*n =* 23,959)
True positive screens, *n* (%)	0 (0)	5 (0.02)
False negative screens, *n* (%)	0 (0)	5 (0.02)
False positive screens, *n* (%)	32 (1.0)	221 (0.9)
Respiratory pathology	8	88
Infection/sepsis	3	31
Non-critical CHD	3	3
Other pathology	2	12
Healthy	16	87
